# *Atractylodes chinensis* polysaccharides downregulate RHEB to confer splenoprotective effects against aging-associated disorders

**DOI:** 10.3389/fphar.2026.1877665

**Published:** 2026-08-06

**Authors:** Xiangyan Tu, Yang Liu, Kaiting Yang, Chunhan Qiao, Yidan Chen, Zhumeng Liu, Shuai Ma, Yingzhe Li, Tian Zhang, Yu Cao, Chunying Zhao

**Affiliations:** 1 Hebei Province Key Laboratory of Research and Development of Traditional Chinese Medicine, Institute of Chinese Materia Medica, Chengde Medical University, Chengde, Hebei, China; 2 Hebei Province Key Laboratory of Nerve Injury and Repair, School of Basic Medical Sciences, Chengde Medical University, Chengde, Hebei, China; 3 Department of Cardiology, Second Medical Center, Chinese PLA General Hospital, Beijing, China; 4 State Key Laboratory for Quality Ensurance and Sustainable Use of Dao-di Herbs, National Resource Center for Chinese Materia Medica, China Academy of Chinese Medical Sciences, Beijing, China; 5 Hebei Province Academy of Chinese Medicinal Materials Industrial Technology, Institute of Chinese Materia Medica, Chengde Medical University, Chengde, Hebei, China

**Keywords:** aging, spleen, *Atractylodes chinensis* polysaccharides, RHEB, lymphoma

## Abstract

**Background:**

Population aging represents a major global health challenge, highlighting the need for interventions that target fundamental aging mechanisms. The spleen, a central organ in both traditional medicine and systemic immunity, undergoes functional decline with age.

**Methods:**

The present study investigated the splenoprotective effects and underlying mechanisms of *Atractylodes chinensis polysaccharides* (ACP) in aging from the perspective of targeted regulation.

**Results:**

In aged mice, ACP treatment attenuated splenic apoptosis and decreased the expression of the pro-inflammatory mediators TNFAIP2 and CXCR3. Integrative proteomic and transcriptomic profiling identified the small GTPase RHEB as a critical regulatory hub. Mechanistically, ACP treatment downregulated expression of RHEB involved to the mTORC1 pathway with notable statistical significance at the protein level. Structural and molecular docking analyses revealed high cross-species conservation of RHEB and stable binding between ACP monosaccharides and both RHEB and specific E3 ubiquitin ligases RNF185 and RNF121. The molecular glue property further accounted for the downregulation of RHEB protein, which is mediated by ubiquitination and degradation. *In vitro*, ACP treatment mirrored the effects of RHEB knockdown, resulting in reduced reactive oxygen species accumulation and senescence-associated β-galactosidase activity. Integrative analysis between proteomics data and clinical lymphoma data of spleen revealed that ACP reversed the age-associated expression of genes linked to senescence and tumorigenesis (*ASPM, SKA1*, and *FOXO1*).

**Conclusion:**

Collectively, these findings indicate that ACP alleviates splenic aging by targeting RHEB and modulating apoptosis, inflammatory events, oxidative stress, and the cellular senescence phenotype, thereby providing a mechanistic foundation for developing ACP-based strategies to protect the spleen through delayed aging.

## 1 Introduction

Population aging has become a defining global demographic shift. By 2050, the proportion of the world’s population aged over 60 is projected to double to 22%, with 80% of older adults living in low- and middle-income countries (Ding et al., 2024). The period from 1990 to 2023 witnessed a more than fivefold increase in the absolute disease burden among the oldest old, largely attributable to ischemic heart disease, stroke, chronic kidney disease, Alzheimer’s disease and other dementias (Tang et al., 2026). Concurrently, the incidence of cancer increases markedly with aging, and the two processes converge along hallmark axes, including inflammation-immune imbalance and cellular senescence (Liang et al., 2026). Senescent cells acquire a senescence-associated secretory phenotype (SASP), characterized by the release of pro-inflammatory cytokines, chemokines, growth factors, and matrix-remodeling enzymes (Kusmanto, 2025). While senescence initially serves as a barrier against malignant proliferation, the persistent accumulation of senescent cells and their SASP-driven chronic inflammation create a pro-tumorigenic microenvironment that promotes cancer initiation, progression, and therapy resistance (Kusmanto, 2025). Thus, targeting the shared mechanistic nodes between aging and cancer represents a promising strategy to simultaneously mitigate age-related pathologies and improve oncological outcomes.

The importance of the spleen lies in its role as the body’s largest secondary lymphoid organ and blood filter, undertaking indispensable physiological functions including immune defense, quality control of red blood cells, regulation of hematopoiesis, and serving as a reservoir for blood and immune cells (Ladu and Hibbs, 2025). Notably, approximately 5% of blood volume passes through the spleen with each cardiac cycle, positioning it as a sentinel organ that constantly surveys circulating antigens and pathogens (Tubman, 2024). This unique anatomical and physiological feature renders the spleen a highly sensitive barometer of systemic health and age-related changes. The effects of aging on the spleen are characterized by structural degeneration, remodeling of immune cell composition, and functional decline. These manifest as disorganization of splenic microanatomy, reduced capacity of the red pulp to clear senescent red blood cells, impaired germinal center reactions and T-B cell collaboration in the white pulp, weakened ability of the marginal zone to capture blood-borne antigens, as well as adaptive changes in T cells that prioritize survival over function, induced by iron/heme deposition in the splenic microenvironment, ultimately leading to increased susceptibility to infections, diminished vaccine responsiveness, and exacerbated systemic immunosenescence (Jang et al., 2026; Ezuz et al., 2025; Bellomo et al., 2021). Given that the spleen integrates both innate and adaptive immunity and is directly exposed to age-induced inflammatory and metabolic insults through the bloodstream, it serves as an ideal sentinel organ for investigating systemic aging and evaluating potential anti-aging interventions.

Natural plant polysaccharides are increasingly recognized as important food metabolites that retard aging (Deng et al., 2023). Their antiaging properties are mediated by regulating apoptosis, modulating gene expression, improving mitochondrial dysfunction, scavenging free radicals, increasing telomerase activity, enhancing immunity, activating autophagy, inhibiting glycation, and modulating the gut microbiota (Deng et al., 2023). *Atractylodes* is a genus of medicinal plants native to China, Korea, and Japan (Kim et al., 2016), and among its species, *Atractylodes chinensis* is utilized as a botanical raw material for health foods (Gao et al., 2023). Dried rhizomes derived from *Atractylodes chinensis* exhibit the functions of strengthening the spleen, brightening the eyes, treating diarrhea and arthritis (Gao et al., 2023; Hu et al., 2025). The cream containing *Atractylodes chinensis* can slow skin aging (Im et al., 2022), and *Atractylodes chinensis* polysaccharides (ACP) can reverse oxidative stress-induced autophagy inhibition in HaCaT cells (Wang et al., 2025). In addition, the polysaccharide of *Atractylodes macrocephala* Koidz [Asteraceae; Atractylodis macrocephalae rhizoma] known as an immune enhancer ameliorates cyclophosphamide-induced splenic injury, T- and B-cell proliferation suppression, leukocyte dysregulation, and impairment of both humoral and cellular immunity (Li et al., 2018). Whereas, the role of ACP in the spleen, especially its targeted regulatory effects against splenic aging and the underlying mechanisms, remains unknown.

Despite the recognized anti-aging properties of natural plant polysaccharides and the documented protective effects of *Atractylodes macrocephala* polysaccharide against cyclophosphamide-induced splenic injury, the specific role of ACP in combating splenic aging remains completely unknown. This knowledge gap is significant because the spleen undergoes profound structural degeneration, immune cell remodeling, and functional decline with age, in which processes drive systemic immunosenescence, increase infection susceptibility, and blunt vaccine responses (Liu et al., 2025a; Chen et al., 2024), yet no targeted botanical drugs have been established to mitigate splenic aging. Although ACP has been shown to reverse oxidative stress-induced autophagy inhibition in HaCaT cells (Wang et al., 2025), its direct effects on the aged spleen and the underlying molecular mechanisms have never been investigated. Besides, aging is a complex biological process governed by multi-factor coordination. Single-layer omics data are insufficient to delineate global systemic changes, and no multi-omics study has yet been applied to characterize ACP’s splenoprotective actions. Therefore, the present study adopted a multi-omics framework (integrating proteomics, transcriptomics, and machine-learning-driven analysis) to systematically uncover the mechanisms underlying ACP-mediated splenoprotective potency in aging. Specifically, the present study aimed to answer (i) whether ACP supplementation ameliorates aging-induced splenic apoptotic and inflammatory responses; (ii) which key moleculars are targeted by ACP in the aged spleen; and (iii) how ACP attenuates splenic aging through targeted molecular intervention.

## 2 Materials and methods

### 2.1 ACP acquisition

The polysaccharides from Atractylodes chinensis (Bunge) Koidz. [Asteraceae; Atractylodes chinensis rhizoma] used in this study were provided by the Hebei Province Key Laboratory of Research and Development of Traditional Chinese Medicine (Chengde, China). It was from the same batch (No. 20240727) and prepared according to the protocol previously described by our group (Tu et al., 2025). Briefly, crude ACP was extracted by ultrasonic-assisted extraction, followed by deproteinization using the Sevag method and decolorization with macroporous resin. After acid hydrolysis, the monosaccharides were derivatized with 1-phenyl-3-methyl-5-pyrazolone (PMP) and analyzed by high-performance liquid chromatography. The purity of ACP was determined to be 92.08% using the phenol-sulfuric acid method with UV-Vis spectrophotometry UV-2600 (Shimadzu, Japan) at 490 nm. The contents of ACP metabolites were determined using both the Quantitative Analysis of Multicomponents by a Single Marker and the External Standard Method. Analysis revealed that ACP consisted of glucuronic acid, anhydrous glucose, galactose, and arabinose in a mass ratio of approximately 2.85 : 1 : 1.28 : 2.62 (Tu et al., 2025).

### 2.2 Animal experiments and sampling

**FIGURE 1:**
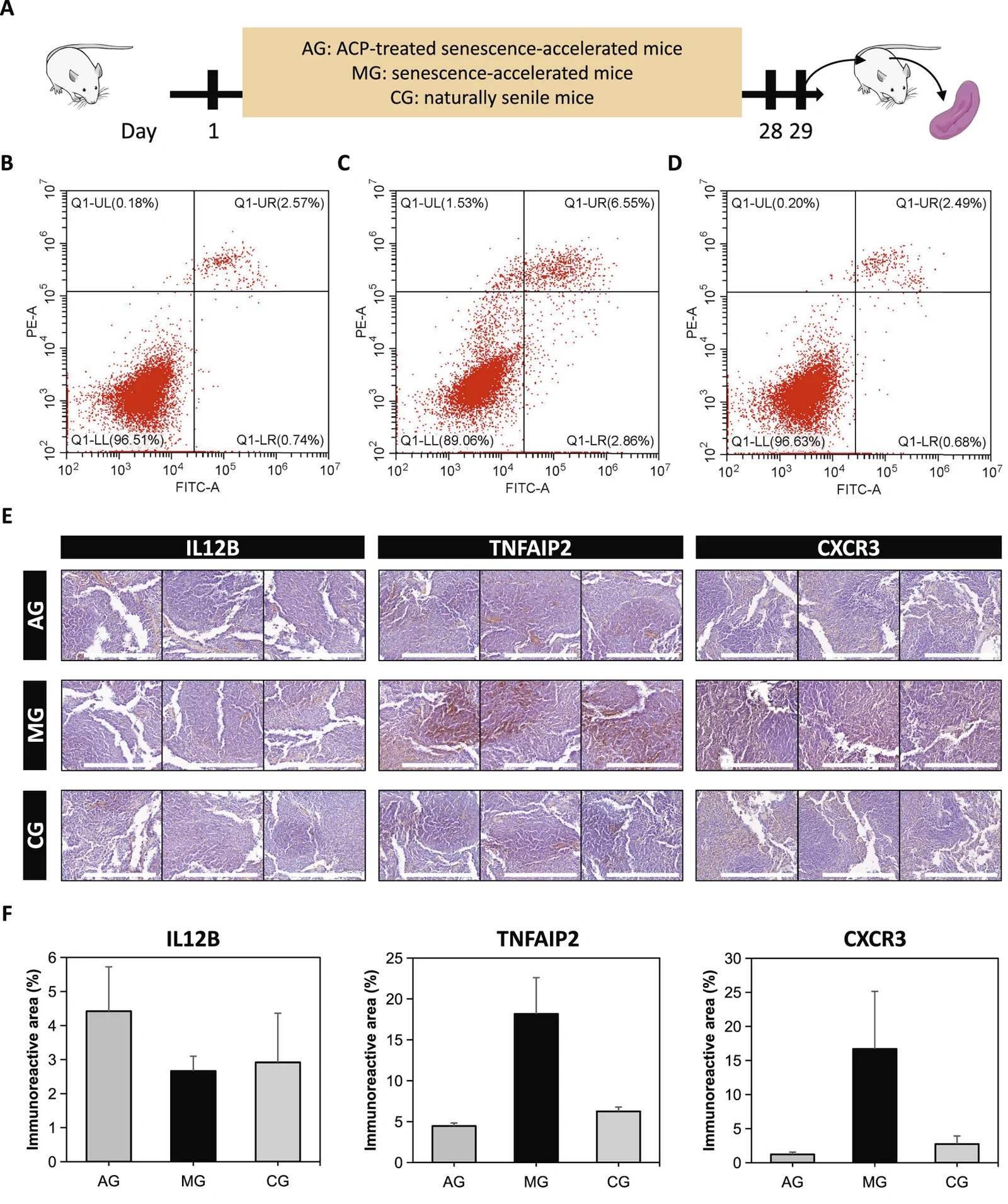
Anti-aging effect evaluation of ACP. **(A)** Schematic diagram of the experimental procedure. **(B–D)** Apoptotic cell proportion in spleen tissue from **(B)** AG **(C)** MG, and **(D)** CG. **(E)** Analysis of inflammation-related factors of spleen tissue in the scale of 100 μm. **(F)** Immunoreactive area quantification for IL12B, TNFAIP2, and CXCR3. AG, MG, and CG severally indicate ACP-treated group, aging model group and control group.

The experimental cohort comprised 45 six-month-old specific pathogen-free (SPF) mice, with 15 mice per group (Nakamura et al., 2014; Xie et al., 2024; Dacomo et al., 2024; Liu et al., 2025b). Thirty senescence-accelerated mouse prone 8 (SAMP8) mice (38.40 ± 1.92 g) were assigned to either an ACP-treated group (AG) or an aging model group (MG), with naturally senile 15 C57BL/6 wild-type mice (27.98 ± 2.09 g) serving as a healthy control group (CG). The ACP-treated SAMP8 mice (n = 15) received an ACP suspension via oral gavage at a dose of 200 mg/kg (Zeng et al., 2018). The aging model group (n = 15) was administered 10 mL/kg of double-distilled water by gavage. The C57BL/6 wild-type controls (n = 15) were housed under standard conditions (22 °C, 12-h light/dark cycle) with *ad libitum* access to food and water. The experiment lasted for 28 days (Figure 1A). Following anesthesia with Zoletil 50 (75 mg/kg), all 45 mice from the three groups were euthanized by exsanguination on day 29. Spleen tissues were collected, placed into 1.5 mL cryotubes, and promptly stored at −80 °C for subsequent analysis.

### 2.3 Apoptosis detection by flow cytometry

Spleen tissues from four samples per group were reserved for apoptosis analysis, with three samples allocated for preliminary optimization of cell yield and one sample used for formal experimentation. Tissues were minced and digested with collagenase type II and trypsin. The resulting cell suspension was filtered through a cell strainer to remove debris. Cells were collected by centrifugation, washed with ice-cold phosphate-buffered saline (PBS), and resuspended in 500 μL of binding buffer at a concentration of 1 × 10^7^ cells/mL. A 100-μL aliquot of the cell suspension was transferred to a 5 mL flow tube and stained using an Annexin V-FITC/PI Apoptosis Detection Kit (BioLegend, United States) according to the manufacturer’s instructions. After incubation in the dark at room temperature for 15 min, samples were analyzed on a CytoFLEX flow cytometer (Beckman Coulter, United States).

### 2.4 Immunohistochemistry of spleen tissue

Paraffin-embedded spleen sections (one sample per group) were heated at 60 °C for 45 min, deparaffinized in xylene (two changes, 10 min each), and rehydrated through a graded ethanol series (100%, 95%, 85%, and 75%) followed by a 5 min rinse in distilled water. Antigen retrieval was performed by incubating sections in citrate buffer (pH 6.0) with heat treatment for 8 min, after which slides were cooled to room temperature and washed with 0.01 M PBS (pH 7.4). Endogenous enzyme activity was quenched by treatment with 3% H_2_O_2_ for 10 min at room temperature, followed by additional PBS washes. Sections processed by 5% goat serum were incubated with diluted primary antibody at 4 °C overnight. Slides were rewarmed at 37 °C for 30 min, washed in PBS, and incubated with 60 μL of secondary antibody for 30 min at 37 °C. After further PBS washes, immunoreactivity was visualized using DAB Chromogenic Reagent Kit (Servicebio, China). Sections were rinsed in distilled water, counterstained with hematoxylin for 2 min, and rinsed again. Finally, the sections were dehydrated through a graded ethanol series, cleared in xylene (two changes, 10 min each), mounted with neutral balsam, and imaged under a DM2000 LED microscope (Leica, Germany).

### 2.5 Protein extraction and data-independent acquisition (DIA) mass spectrometry

Proteins from spleen tissues (five samples per group; three groups in total) were extracted using a lysis buffer containing 8 M urea and 1% SDS. The procedure included homogenization using a high-flux tissue grinding machine (three cycles, 180 s each), non-contact cryogenic sonication for 30 min, and centrifugation at 16,000 *g* for 30 min at 8 °C. Protein concentration was determined using a BCA Protein Assay Kit (Thermo Scientific, United States). Following protein digestion and peptide desalting, peptide quantification was performed on a NanoDrop One system (Thermo Scientific, United States) by measuring UV absorbance. Based on the quantification results, peptides were analyzed using a Vanquish Neo UHPLC system coupled to an Orbitrap Astral mass spectrometer (Thermo Scientific, United States). DIA raw data were processed with Spectronaut v19 using the following parameters: peptide length = 7–52; enzyme = trypsin/P; maximum missed cleavages = 2; fixed modification = carbamidomethylation (C); variable modifications = oxidation (M) and N-terminal acetylation; Protein FDR ≤0.01; Peptide FDR ≤0.01; Peptide Confidence ≥99%; XIC width ≤75 ppm (Kohler et al., 2024). Protein quantification was performed using the MaxLFQ algorithm (Cox et al., 2014).

### 2.6 RNA extraction, library preparation, and sequencing

Total RNA was extracted from the spleen tissues of three groups (five samples per group) using TRIzol Reagent. RNA integrity and concentration were assessed with an Agilent 5300 Bioanalyzer (Agilent, United States) and a NanoDrop 2000 system (Thermo Scientific, United States), respectively. Sequencing libraries were constructed from 1 μg of total RNA using the Illumina Stranded mRNA Prep Ligation Kit (Illumina, United States) according to the manufacturer’s protocol. The resulting libraries were sequenced on an Illumina NovaSeq X Plus platform with a NovaSeq Reagent Kit (Illumina, United States). Raw reads were processed with fastp (under default parameters) for adapter trimming and quality control to obtain clean reads (Chen et al., 2018). These clean reads were then aligned in orientation-aware mode to the *Mus musculus* reference genome using HISAT2 (Kim et al., 2015). Transcript assemblies were generated for each sample using StringTie (Pertea et al., 2015).

### 2.7 Bioinformatics analysis of proteins and transcripts

Proteins from the two comparison groups were analyzed for statistical significance and fold change using a t-test implemented in R. Differentially expressed proteins (DEPs) were defined as those with a p-value <0.05. For transcriptomic analysis, gene abundance was quantified with RSEM based on gene count (Li and Dewey, 2011), and differentially expressed genes (DEGs) was assessed using DESeq2 and limma (Love et al., 2014; Ritchie et al., 2015). Weighted gene co-expression network analysis (WGCNA) and correlation analysis were subsequently performed to explore co-expression patterns and relationships. The signaling pathway through which ACP delays spleen senescence was constructed based on DEPs using the OMICSEE platform (Li et al., 2025a). The terminal effector proteins in this pathway stitched up the corresponding core regulatory genes that exhibited consistent expression patterns at both the protein and mRNA levels.

### 2.8 Molecular docking analysis of ACP-target interactions

To assess the translational relevance of the identified core regulatory genes, Evo Designer and UniProt-Align were utilized to align their DNA and protein sequences between mice and humans, respectively (Ahmad et al., 2025; Nguyen et al., 2024). Molecular dynamics simulations were then performed for the corresponding core proteins with CABSflex 2.0 and iMODS to evaluate their structural flexibility and dynamic behavior (Kuriata et al., 2018; López-Blanco et al., 2014). DockSurf in Mobyle was used to predicted possibility of ligand-protein conformation (Barbault et al., 2023). Evolutionary conservation of the core proteins was analyzed with ConSurf (Glaser et al., 2003). For molecular docking, the three-dimensional structures of four ACP metabolites (glucuronic acid, anhydrous glucose, galactose, and arabinose) were retrieved as SDF-formatted conformers from PubChem, while the protein structures were obtained from the Protein Data Bank (PDB). The interactions between ACP metabolites and core proteins were validated using Discovery Studio (Li et al., 2025b), CB-Dock2 (Liu et al., 2022a), and SwissDock (Bugnon et al., 2024). The core protein corresponding to ubiquitinase was clarified by UniProt and UbiBrowser 2.0 (Wang et al., 2022).

### 2.9 Cell culture and small RNA interference

DMEM/F-12 medium containing 10% fetal bovine serum and 1% bispecific antibody was used to culture HK-2 cells in a 37 °C, saturated humidity, 5% CO_2_ incubator. Logarithmic-phase HK-2 cells were trypsinized (0.25%) and seeded in 6-well plates at 5 × 10^5^ cells/well for subsequent culture. For sRNA-mediated gene suppression, the small RNA oligos with attached GP-siRNA-Mate Plus reagent were purchased from Uptbio (China) and synthesized as small interfering RNA (siRNA). Using liposome 2000, the siRNA-RHEB (sense: 5′-CCG​GGC​AAG​AUG​AAU​AUU​CUA​TT-3’; antisense: 5′-UAG​AAU​AUU​CAU​CUU​GCC​CGG​TT-3′) targeting human-derived RHEB mRNA and si-NC (sense: 5′-UUC​UCC​GAA​CGU​GUC​ACG​UTT-3’; antisense: 5′-ACG​UGA​CAC​GUU​CGG​AGA​ATT-3′) acting as negative control were independently transfected into HK-2 cells, a reliable aging-related model *in vitro* (Rattananinsruang et al., 2023). The control group was the blank condition without the addition of any small interfering RNA. RNAs were extracted by TRIzol Reagent from HK-2 cells. The SnapGene was used to design primers (RHEB-F: 5′-TTG​TTG​GAT​ATG​GTG​GGG​AAA​G-3’; RHEB-R: 5′-CAT​CAC​CGA​GCA​TGA​AGA​CTT-3’; β-actin-F: 5′-CCC​TGG​AGA​AGA​GCT​ACG​AG-3’; β-actin-R: 5′-CGT​ACA​GGT​CTT​TGC​GGA​TG-3′). Following reverse transcription by PerfectStart Green qPCR SuperMix (TransGen, China), quantitative real-time PCR (qRT-PCR) was performed on the QuantStudio 1 (Applied Biosystems, USA).

### 2.10 Flow cytometric detection of reactive oxygen species

HK-2 cells were divided into four experimental groups, including ACP (treated with ACP), NC (ACP-free), si-RHEB (transfected with si-RHEB), and si-NC (transfected with non-targeting siRNA). Cells from each group were collected, washed with ice-cold PBS, and resuspended in 500 μL of binding buffer at a density of 1 × 10^7^ cells/mL. A 100 μL aliquot of the cell suspension was transferred to a 5 mL flow cytometry tube. Intracellular reactive oxygen species (ROS) levels were assessed using a ROS Assay Kit (Beyotime, China) according to the manufacturer’s instructions, and samples were analyzed on a CytoFLEX flow cytometer (Beckman Coulter, United States).

### 2.11 Senescence-associated β-galactosidase assay

Following removal of the culture medium and washing with PBS, cellular senescence of HK-2 cells was assessed in the ACP, NC, si-RHEB, and si-NC groups using a Senescence β-Galactosidase Staining Kit (Beyotime, China) according to the manufacturer’s instructions. After overnight incubation at 37 °C, stained cells were visualized and quantified under a DS-Fi3 microscope (Nikon, Japan).

### 2.12 Clinical data acquisition and analysis of spleen aging-associated diseases

Transcriptomic datasets related to clinical manifestations of splenic dysfunction were obtained from the NCBI GEO database (accessions: GSE57520 and GSE25550). Differential expression analysis was performed using GEO2R (Barrett et al., 2013), with genes defined as differentially expressed if they satisfied p < 0.05. Differentially expressed genes were functionally annotated to Gene Ontology (GO) and Kyoto Encyclopedia of Genes and Genomes (KEGG) pathway databases by Metascape (Zhou et al., 2019). Aging- or anti-aging-related genes were severally retrieved from the GeneCards human genome database using the search term “aging” or “anti-aging”. Finally, correlation analyses were conducted by integrating proteomic and transcriptomic data from spleen tissues with the clinical transcriptomic datasets.

### 2.13 Statistical analysis

In addition to the negative binomial model used for transcriptome sequencing data, the t. test function in R was used to perform a t-test for comparisons between groups. Results were expressed as mean ± SD, and statistical significance was set at p < 0.05.

## 3 Results

### 3.1 Attenuation of age-related splenic apoptosis and inflammation by ACP

Apoptosis in spleen tissue was assessed by flow cytometry. The total apoptotic rates were 3.31% in AG, 9.41% in MG, and 3.17% in CG (Figures 1B–D), indicating that ACP treatment attenuated apoptosis. Inflammation was evaluated by immunohistochemical staining of the pro-inflammatory markers IL12B, TNFAIP2, and CXCR3. In contrast to IL12B, strong staining intensities of TNFAIP2 and CXCR3 were showed in MG than AG and CG (Figure 1E). Differ from high-proportion IL12B in AG, the positive area percentages for TNFAIP2 were 4.462% (AG), 18.166% (MG), and 6.256% (CG), and for CXCR3 were 1.215% (AG), 16.704% (MG), and 2.739% (CG) (Figure 1F). Accordingly, the lowest level of positive staining (brown DAB signal) for both markers was observed in the AG group, which mirrored the quantified area percentages.

### 3.2 Multi-omics profiling RHEB as a central regulator of ACP-delayed spleen aging

**FIGURE 2:**
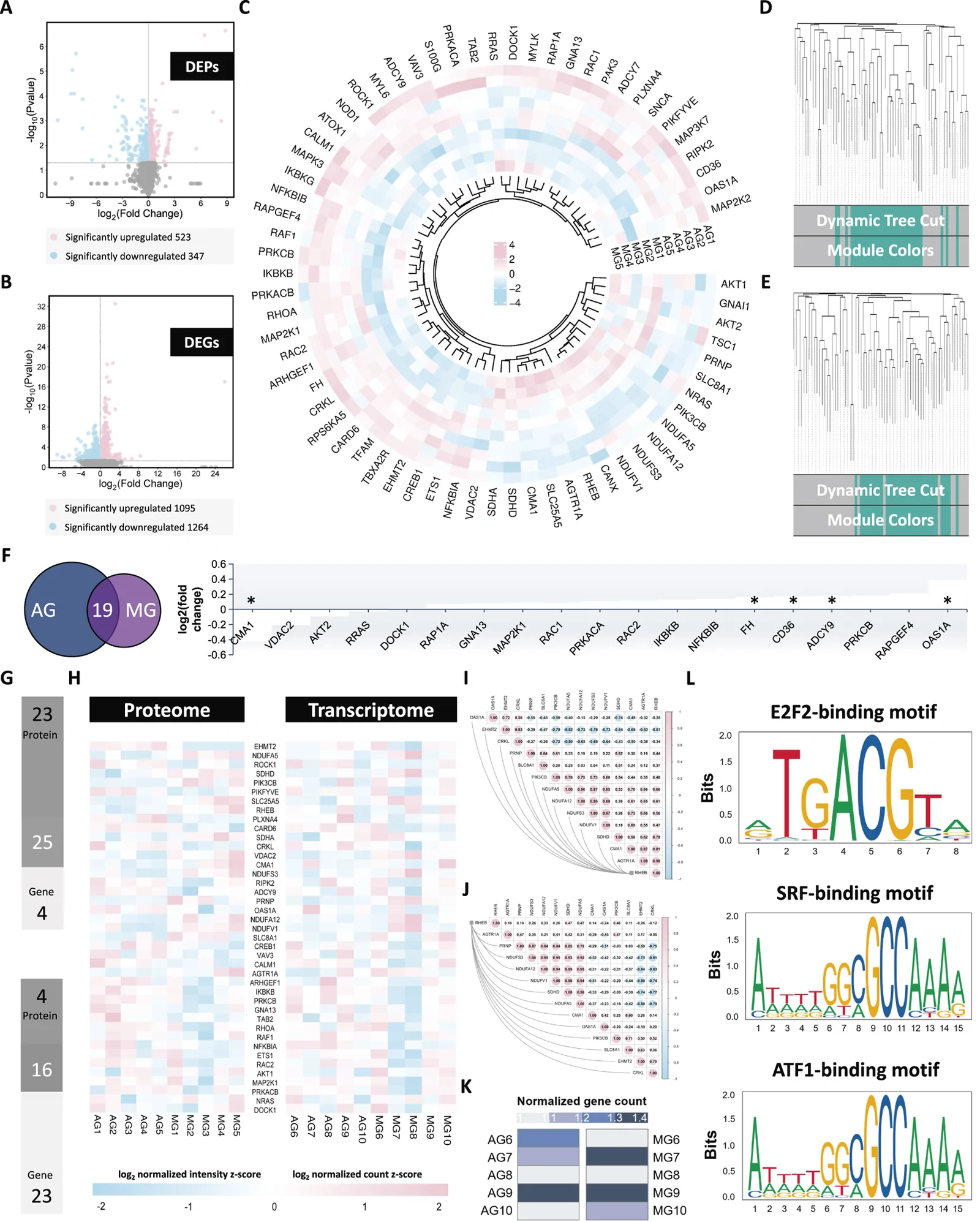
Multi-omic analysis of ACP-mediated anti-aging effects. **(A)** Differentially expressed proteins (DEPs) and **(B)** differentially expressed genes (DEGs) between the AG and MG. **(C)** Expression levels of hub proteins within the ACP-mediated anti-aging signaling pathway **(D,E)** WGCNA of hub proteins in **(D)** AG and **(E)** MG. **(F)** Log_2_(Fold Change) values of commonly co-expressed hub proteins between AG and CG (*p < 0.05). **(G)** Number of common upregulated (top) and downregulated (bottom) hub proteins/genes comparing AG to MG. **(H)** Expression levels of the hub proteins/genes shown in **(G)**. **(I,J)** Correlation analysis among **(I)** hub proteins and **(J)** their corresponding genes. **(K)** RHEB gene expression levels across samples. **(L)** Motif sequences of three RHEB transcription factors (E2F2, SRF, and ATF1). AG, MG, and CG indicate ACP-treated group, aging model group and control group, respectively.

**FIGURE 3:**
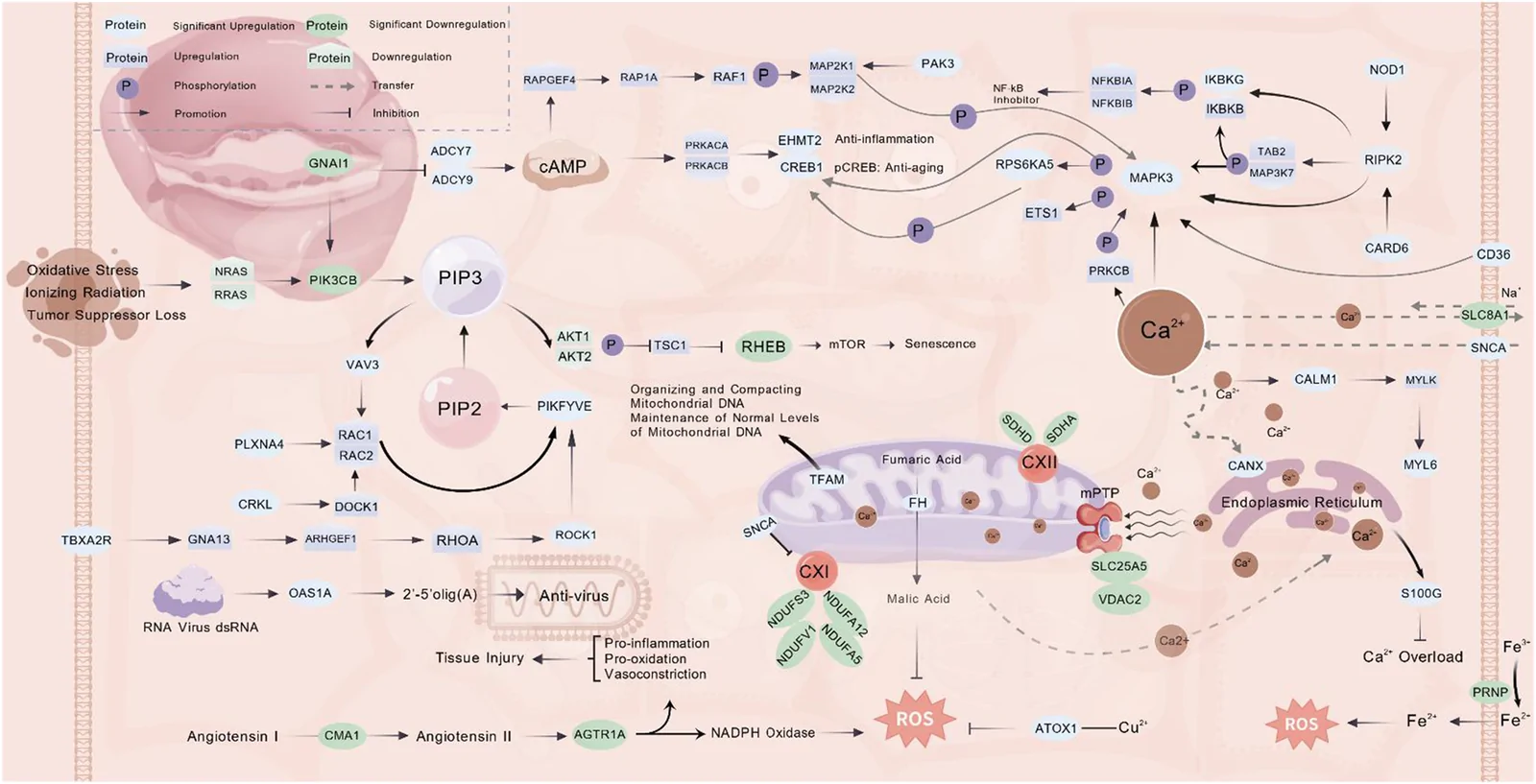
ACP-mediated signaling pathway counteracts aging in the spleen.

Statistical analysis revealed significant differences between the AG and MG in spleen tissues (p < 0.05), with 870 DEPs (523 upregulated and 347 downregulated) and 2359 DEGs (1095 upregulated and 1264 downregulated) (Figures 2A,B). Using local OMICSEE platform, the signaling pathway mediated by ACP was profiled by 68 DEPs (e.g., CREB1, EHMT2, and RHEB) coupled to resist aging in spleen (Figures 2C, 3). WGCNA highlighted co-expression module comprising 19 DEPs common both in AG and MG (Figures 2D–F). Based on integrated proteomic and transcriptomic analyses (Figures 2G,H), the terminal effector RHEB was identified as a central anti-aging factor. RHEB was positively correlated with significantly downregulated (p < 0.05) CMA1 (r_protein_ = 0.81, r_transcript_ = 0.60), SLC8A1 (r_protein_ = 0.37, r_transcript_ = 0.77), PIK3CB (r_protein_ = 0.48, r_transcript_ = 0.80) associated with pro-aging (Figures 2I,J). Under ACP intervention, RHEB protein expression was significantly downregulated (p < 0.05), while its transcript level showed a consistent decreasing trend (Figures 2H,K). Furthermore, the significantly reduced (p < 0.05) transcription factors E2F2, SRF, and ATF1 at protein levels were recognized for regulating RHEB transcription through binding motif sequence of RHEB promoter (Figure 2L).

### 3.3 Cross-species conservation and stable conformation of RHEB

**FIGURE 4:**
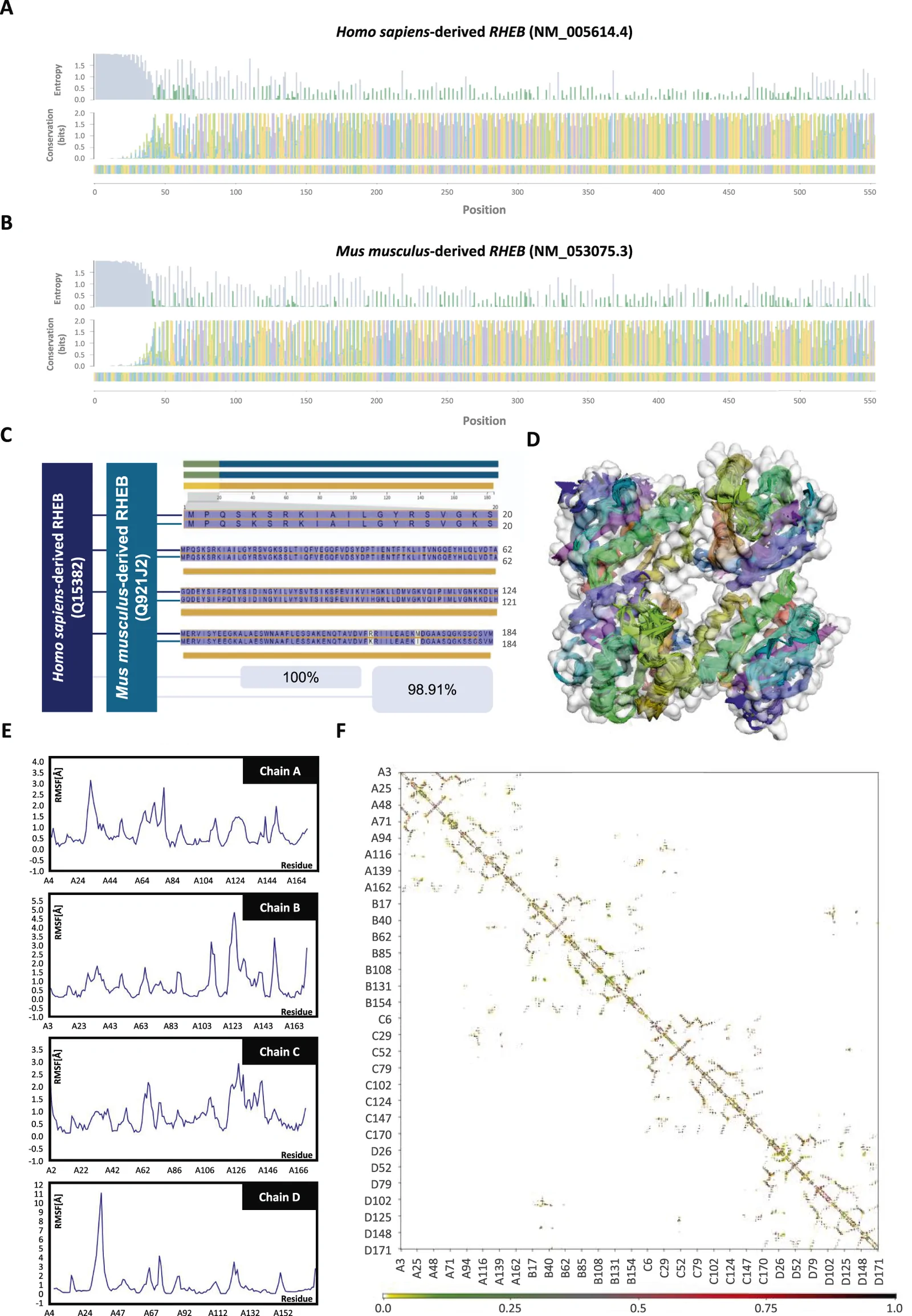
Cross-species analysis of RHEB. **(A,B)** Gene sequence conservation analysis of RHEB in **(A)** *Homo sapiens* and **(B)** *Mus musculus*. **(C)** Protein sequence similarity between human and mouse RHEB. **(D)** Predicted structure of RHEB protein. **(E)** RMSF of amino acid residue across different RHEB chains. **(F)** Contact map of the residues of RHEB.

**FIGURE 5:**
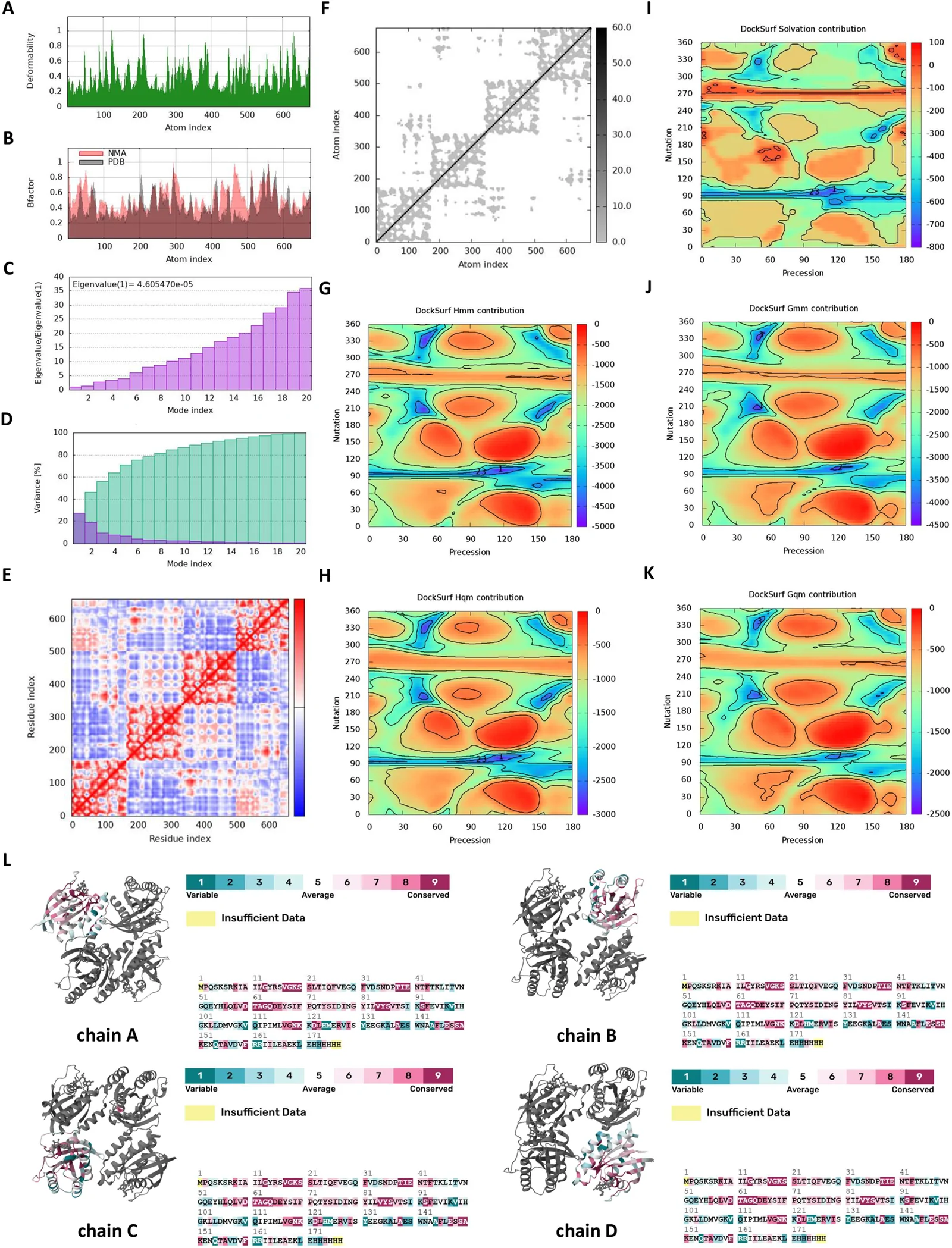
Molecular dynamics simulation of RHEB. **(A)** Main-chain deformability. **(B)** B-factors values. **(C)** The eigenvalue. **(D)** Variance. **(E)** Co-variance map. **(F)** Elastic network model representation. **(G–K)** Thermodynamic profiles on **(G)** ΔH_MM_, **(H)** ΔH_QM_, **(I)** ΔSolv, **(J)** ΔG_MM_, and **(K)** ΔG_QM_. **(L)** Protein sequence conservation across different chains.

DNA sequences corresponding to RHEB were conserved in *Homo sapiens* and *Mus musculus* (Figures 4A,B), sharing 98.91% amino acid identity (Figure 4C). The structural model of human-derived RHEB (PDB: 7BTC) comprised four chains (A-D) (Figure 4D). Root-mean-square fluctuation (RMSF) analysis indicated that most active residues fluctuated within 2.0 Å across all chains, reflecting overall structural stability (Figure 4E). Analysis of the residue contact network within RHEB revealed regions of mild-intensity contact, which correspond to potential binding interfaces for ACP (Figure 4F). Deformability analysis revealed limited hinge motion (≤1 Å) (Figure 5A), while B-factor calculations highlighted regions of higher atomic displacement (Figure 5B). The eigenvalue for RHEB was 4.605,470 × 10^−5^ with variance matrix of residues (Figures 5C,D). Residue covariance matrices visualized correlated (red), uncorrelated (white), and anti-correlated (blue) motions (Figure 5E), and the elastic network model illustrated interatomic connectivity, with stiffer springs depicted in darker regions (Figure 5F). DockSurf analysis further identified multiple low-energy binding landscapes across five thermodynamic maps, including MM enthalpy (ΔH_MM_), QM enthalpy (ΔH_QM_), solvation energy (ΔSolv), MM free energy (ΔG_MM_), and QM free energy (ΔG_QM_) (Figures 5G–K). The consistent low-energy regions across mappings suggested credible ligand-RHEB binding conformations. ConSurf analysis indicated evolutionary conservation of residues in the RHEB protein (average score = 5.69; Figure 5L). Collectively, these results supported the structural stability of RHEB and its potential for stable interaction with ACP.

### 3.4 Structural plausibility of RHEB-ACP-E3 ubiquitin ligase interaction supported via docking

**FIGURE 6:**
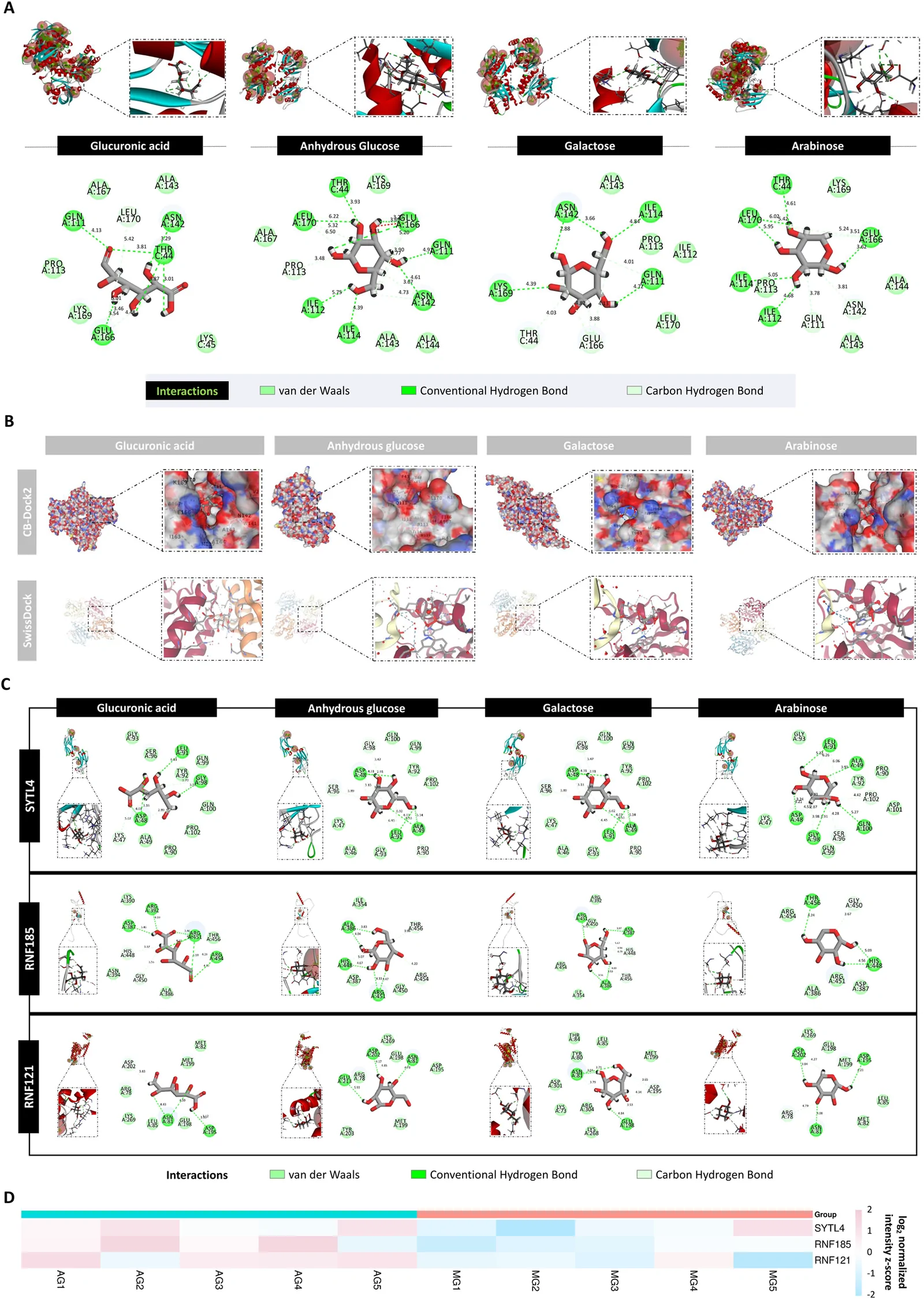
Molecular docking analysis of the RHEB-ACP-E3 ligase system. **(A)** Binding potential of RHEB with glucuronic acid, anhydrous glucose, galactose, and arabinose, evaluated using Discovery Studio. **(B)** Corresponding docking feasibility for the same ligands with RHEB, predicted by CB-Dock2 and SwissDock. **(C)** Binding potential of E3 ubiquitin ligases with glucuronic acid, anhydrous glucose, galactose, and arabinose, assessed using Discovery Studio. **(D)** Protein expression intensity of three E3 ubiquitin ligases.

Molecular docking was performed to evaluate interactions consistent with the molecular glue hypothesis, assessing four principal ACP monosaccharides (glucuronic acid, anhydrous glucose, galactose, and arabinose) binding to human RHEB (PDB: 7BTC), and to three E3 ubiquitin ligases containing SYTL4 (PDB: 3FDW), RNF185 (PDB: AF-AFQ96GF1F1), and RNF121 (PDB: AF-AFQ9H920F1). For the ACP metabolites, binding energies calculated by Discovery Studio yielded values of −36.666 kcal/mol (glucuronic acid), −36.187 kcal/mol (anhydrous glucose), −34.239 kcal/mol (galactose), and −31.571 kcal/mol (arabinose) with RHEB (Figure 6A). Independent validation using CB-Dock2 gave energies of −6.000, −6.000, −6.200, and −5.600 kcal/mol, respectively, while SwissDock returned values of −4.469, −4.537, −4.398, and −4.529 kcal/mol (Figure 6B). Docking with the E3 ligases using Discovery Studio produced binding energies for SYTL4 of −34.715, −25.940, −30.095, and −23.150 kcal/mol with the four ACP metabolites (Figure 6C). Corresponding values for RNF185 were −29.066, −32.688, −30.484, and −24.408 kcal/mol, and for RNF121 were −26.247, −25.713, −28.434, and −22.750 kcal/mol (Figure 6C). The protein expression levels of RNF185 and RNF121 were significantly higher (p < 0.05) in AG than in MG (Figure 6D). These results aligned with a molecular glue-like mechanism, wherein ACP monosaccharides could simultaneously engage both RHEB and E3 ligases to promote its target-specific ubiquitination, thereby providing mechanistic validation for the aforementioned multi-omics findings.

### 3.5 ACP mimics and RHEB knockdown to alleviate cellular senescence

**FIGURE 7:**
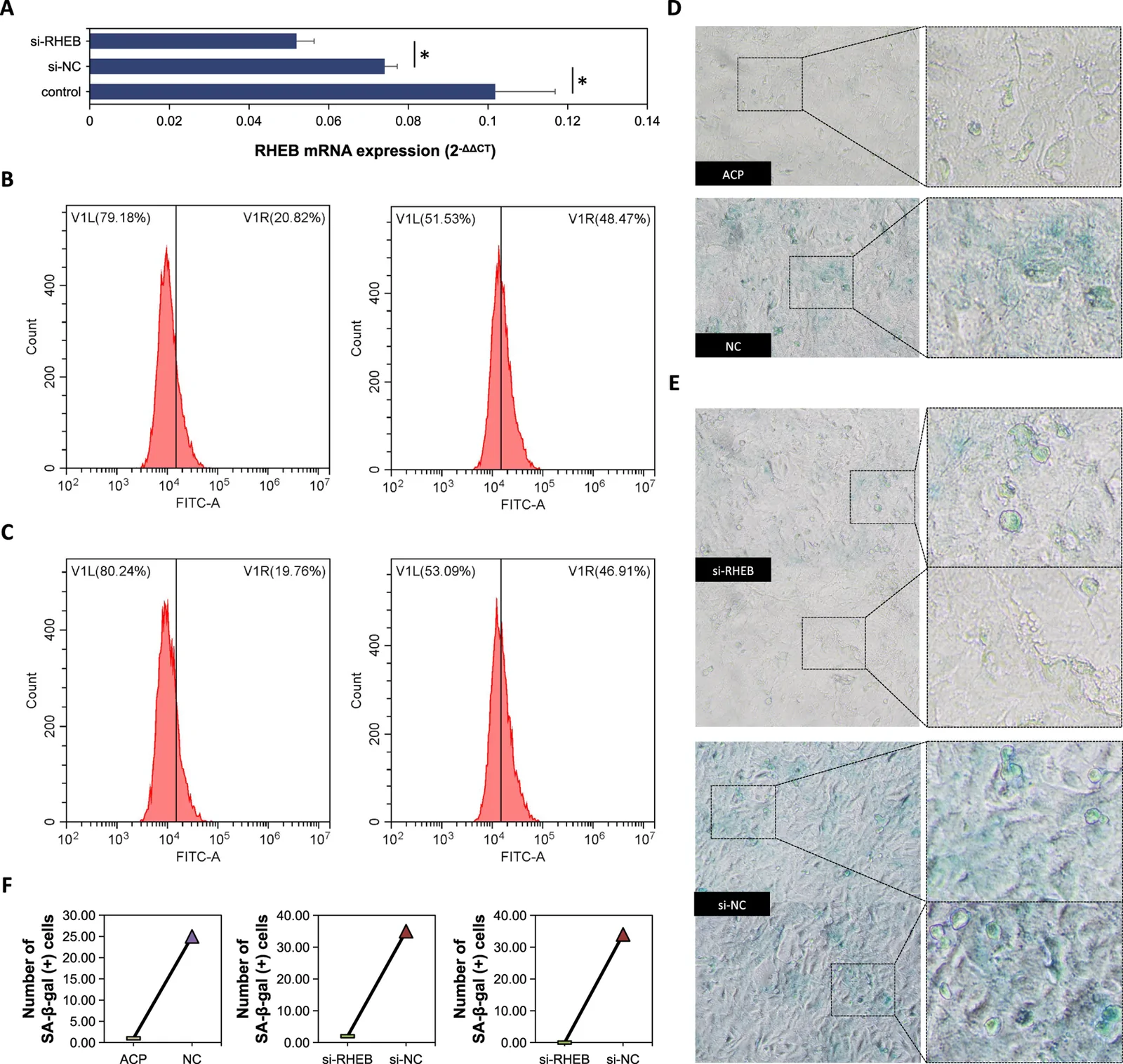
Assessment of anti-aging effects in HK-2 cells. **(A)** RHEB expression following siRNA-mediated knockdown (*p < 0.05). **(B,C)** Intracellular reactive oxygen species levels under **(B)** ACP treatment (left) versus ACP-free (right), and **(C)** si-RHEB (left) versus si-NC (right) **(D,E)** Representative images of SA-β-gal staining (blue). **(F)** Quantification of SA-β-gal-positive cells from multiple microscopic fields (×200 magnification).

The qRT-PCR analysis confirmed that siRNA-mediated knockdown of RHEB significantly reduced its expression compared to both the si-NC and untreated control groups in HK-2 cells (p < 0.05; Figure 7A). Flow cytometry revealed lower levels of ROS in ACP-treated cells (20.82%) than in the NC group (48.47%) (Figure 7B), and similarly in the si-RHEB group (19.76%) compared to the si-NC group (46.91%) (Figure 7C). Senescence-associated β-galactosidase (SA-β-gal) staining revealed weak staining intensity in the ACP and si-RHEB groups, whereas strong staining intensity was observed in the NC and si-NC groups (Figures 7D,E). Quantification confirmed fewer of SA-β-gal-positive cells in the ACP group (1 cell) relative to the NC group (25 cells) (Figure 7F), and in the si-RHEB group (2 cells under microscopic field I and 0 cells under microscopic field II) compared to the si-NC group (35 cells under microscopic field I and 34 cells under microscopic field II) (Figure 7F), indicating that both ACP treatment and RHEB deletion attenuated cellular senescence.

### 3.6 Clinical validation of omics signatures in splenic dysfunction

**FIGURE 8:**
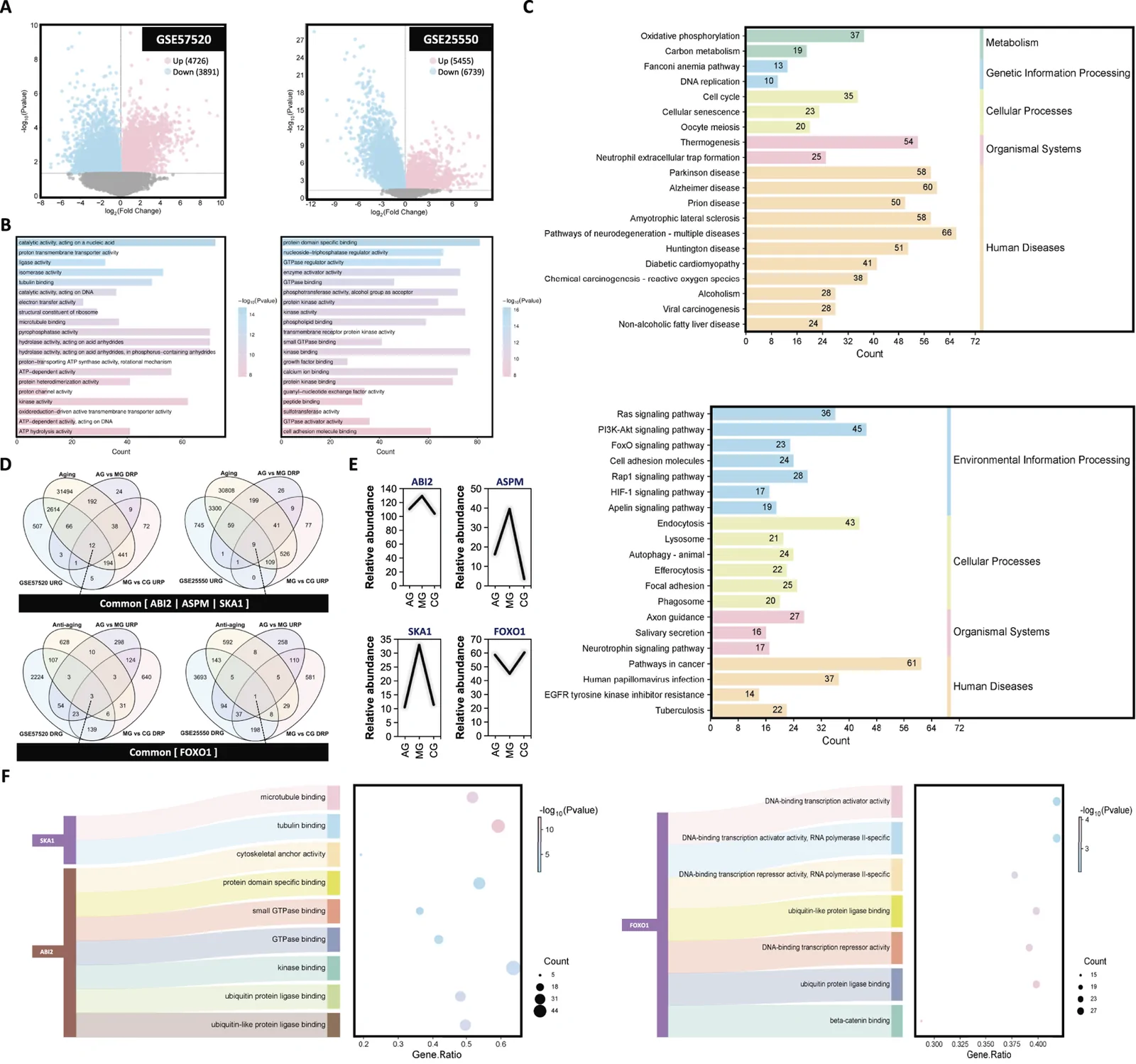
Association of aging-related signatures with splenic pathology. **(A)** Number of differentially expressed genes in spleen transcriptomic datasets GSE57520 and GSE25550 from patients with lymphoma. **(B)** Top 20 enriched molecular function terms from commonly upregulated (left) and downregulated (right) genes in GSE57520 and GSE25550. **(C)** Top 20 enriched KEGG pathways from commonly upregulated (top) and downregulated (bottom) genes in GSE57520 and GSE25550. **(D)** Overlap among aging/anti-aging-related genes, differentially expressed genes in GSE57520 and GSE25550, differentially expressed proteins between AG and MG, and differentially expressed proteins between MG and CG (URG, upregulated gene; DRG, downregulated gene; URP, upregulated protein; DRP, downregulated protein). **(E)** Protein expression levels of ABI2, ASPM, SKA1 and FOXO1 in AG, MG and CG. **(F)** Molecular function terms enriched for ABI2, SKA1 and FOXO1. AG, MG, and CG individually indicate ACP-treated group, aging model group and control group.

To assess the clinical relevance of spleen-related disorders, two public transcriptomic datasets including GSE57520 (spleen tissues: 4 hepatosplenic T-cell lymphoma samples vs. Three normal samples) and GSE25550 (spleen tissues: 14 MALT lymphoma samples vs. 5 normal samples) were collected from the NCBI GEO database. Differential expression analysis identified 8617 and 12,194 significantly dysregulated transcripts in GSE57520 and GSE25550, respectively (p < 0.05; Figure 8A). Genes consistently up- or downregulated across both datasets were subjected to functional enrichment analysis. Commonly upregulated genes were enriched for 307 GO molecular function terms and 112 KEGG pathways, whereas commonly downregulated genes were enriched for 309 GO molecular function terms and 137 KEGG pathways (Figures 8B,C). From the human gene database GeneCards, 35,051 aging-associated genes were retrieved. Cross-referencing with the clinical datasets revealed that three upregulated genes (*ABI2*, *ASPM*, *SKA1*) and one downregulated gene (*FOXO1*) overlapped with the GeneCards anti-aging set (Figure 8D). Following ACP intervention, the protein expression levels of these four genes were significantly reversed (p < 0.05; Figure 8E). Functional annotation of these key members showed that ABI2 was enriched in seven molecular functions (e.g., kinase binding, GTPase binding, small GTPase binding, and protein-domain-specific binding), while SKA1 was enriched in two terms (Figure 8F). FOXO1 was enriched in seven molecular functions (e.g., DNA-binding transcription repressor activity and DNA-binding transcription activator activity) (Figure 8F).

## 4 Discussion

To delineate the mechanism of ACP in delaying spleen aging, the first step constructed a signaling network via bioinformatic analysis of multi-omics data, which pinpointed RHEB as the central target. This identification was consecutively confirmed as cross-species through *in vitro* experiments. Subsequently, an integrative analysis of public GEO clinical data and the present proteomic outcomes showed that ACP intervention reversed the protein levels of ASPM, SKA1, and FOXO1*,* which are three factors that change during aging and are transcriptionally dysregulated in disease samples of the GEO datasets.

### 4.1 Translational relevance of bridging murine to human spleen physiology

Both murine and human spleens share core functions including blood filtration, immune surveillance, and lymphocyte activation, but notable structural variations exist. The spleen of mice exhibits a well-delineated B-cell compartment, the marginal zone, between white and red pulp (Steiniger, 2015). In humans, the marginal zone contains a specialized B cell subpopulation, which recruits and archives memory B cells from immune responses throughout the organism (Kibler et al., 2023). Sheathed capillaries are present in humans but absent in mice (Steiniger, 2015; Kapila et al., 2026). Despite interspecies differences in marginal zone composition and vascular architecture, the fundamental principle of the spleen as a sentinel organ for systemic inflammation is conserved.

### 4.2 *In vivo* evidence of ACP-mediated apoptosis reduction and inflammatory regulation

In healthy individuals, the levels of apoptotic markers increase during aging (Kujoth et al., 2005). The present study found that the splenic apoptotic rate was markedly elevated in aged SAMP8 mice (MG, 9.41%) compared to young C57BL/6 controls (CG, 3.17%), consistent with established age-associated increases in apoptosis. Notably, intervention with ACP reduced the apoptotic rate in treated SAMP8 mice (AG) to 3.31%, a level comparable to that of C57BL/6 mice (CG). These results indicated that ACP could confer protection against age-related cellular damage by attenuating apoptotic activity in the aging spleen. Chronic inflammation is recognized as one of the core hallmarks of aging, which interacts bidirectionally with other hallmarks such as cellular senescence, mitochondrial dysfunction, epigenetic alterations, and disabled autophagy, collectively driving the aging process (Baechle et al., 2023). The pro-inflammatory markers IL12B, TNFAIP2, and CXCR3 are well-established indicators of inflammatory activation (Bhattarai et al., 2026; Xu et al., 2025; Dillemans et al., 2023). The immunohistochemical analysis revealed that TNFAIP2 and CXCR3 were upregulated in the spleen of senescence-accelerated mice compared to young controls, while ACP intervention reduced their expression. These findings provide direct evidence that ACP attenuates age-associated splenic inflammation, at least in part, through coordinated suppression of these two pro-inflammatory mediators. TNFAIP2 (tumor necrosis factor-α-induced protein 2) is a multifunctional protein that promotes inflammation by sustaining NF-κB signaling. Mechanistically, TNFAIP2 binds competitively to KEAP1, thereby preventing the ubiquitin-proteasomal degradation of IKKβ, a key kinase that phosphorylates IκBα and leads to p65 nuclear translocation and transcriptional activation of pro-inflammatory genes (Xu et al., 2025). Thus, the elevated TNFAIP2 expression observed in untreated senescence-accelerated mice likely contributes to sustained NF-κB activity, amplifying the production of inflammatory cytokines and promoting immune dysregulation. The downregulation of TNFAIP2 by ACP suggests that this polysaccharide interrupts this positive feedback loop, thereby reducing NF-κB-driven inflammatory tone. CXCR3 is a chemokine receptor predominantly expressed on activated Th1 cells, CD8^+^ T cells, NK cells, and NKT cells. Its ligands (CXCL9, CXCL10, and CXCL11) are induced by interferon-γ and drive the directional trafficking of these pro-inflammatory lymphocytes into peripheral tissues, including the spleen (Dillemans et al., 2023). The elevation of CXCR3 in the aged spleen therefore reflects an increased pool of inflammatory lymphocytes capable of sustaining local and systemic inflammaging. Notably, ACP treatment reduced CXCR3 expression. Given that CXCR3 is a cell surface receptor that mediates lymphocyte recruitment, its suppression by ACP likely reduces inflammatory cell infiltration into the splenic parenchyma, thereby alleviating the chronic inflammatory burden. In contrast, the upregulation of IL12B was observed following ACP intervention. IL-12 is primarily produced by activated B cells and dendritic cells (Elsner and Shlomchik, 2025), and its increase may reflect the ability of ACP to counteract age-related B cell decline, as previously reported for *Atractylodes* polysaccharides in the spleen (Li et al., 2018). Since B cell production and humoral immunity deteriorate with age (El-Naseery et al., 2020), ACP-enhanced IL12B could represent a beneficial immune-restorative effect rather than a pro-inflammatory event. This dual action suppressing detrimental inflammatory axes while restoring immune vigilance distinguishes ACP from conventional anti-inflammatory agents and highlights its potential as a multi-target intervention against splenic aging.

### 4.3 A multi-omic pathway linking ACP to mTOR inhibition via RHEB

The present study demonstrated that ACP modulated the expression of multiple terminal proteins implicated in aging, including CREB1, EHMT2, and RHEB in the omics-based pathway. Extracellular matrix (ECM) changes have recently been recognized as an indicator of aging, given that a progressive reduction in ECM viscoelasticity occurs systemically during aging, profoundly affecting mitochondrial homeostasis, cellular senescence, and stem cell exhaustion (Kroemer et al., 2025). CREB1, a transcription factor that promotes longevity, directly regulates a broad spectrum of extracellular matrix genes as well as genes encoding matrix remodelers (Vidović and Ewald, 2022). Trained immunity, which represents a form of innate immune memory, has emerged as a potential contributor to chronic inflammation and disease progression (Boden et al., 2026). Induction of trained immunity leads to decreased EHMT2 levels; moreover, pharmacological inhibition of EHMT2 in monocytes amplifies trained immunity responses, as demonstrated by elevated secretion of pro-inflammatory cytokines and an increased metabolic rate (Boden et al., 2026). In this study, ACP intervention significantly upregulated protein expression levels of CREB1 and EHMT2. Notably, dietary restriction, the only intervention proven to extend mammalian lifespan, and downregulation of the mTOR signaling pathway exert a lifespan-extending effect resembling calorie restriction (Campisi et al., 2019; Ortega-Molina et al., 2024). RHEB is a key activator of mTORC1 (Fitzian et al., 2021). RHEB increasing with age induces senescence-associated phenotypes including SA-β-gal in primary MEFs, and the mTORC1 inhibitor rapamycin prevents multiple RHRB-related senescence phenotypic changes (Yeo et al., 2019; Allard et al., 2024). RHEB/mTORC1 mediates oxidative stress such that increasing RHEB expression or enhancing mTORC1 activity eliminates the inhibitory effects on oxidative stress (Li et al., 2025c). In the present work, ACP significantly downregulated RHEB protein expression with reduction of its transcripts, pointing to mTOR inhibition as a central mechanism through which ACP alleviated splenic aging.

### 4.4 Molecular glue-like mechanism underlying ACP-mediated RHEB degradation

Given mTOR’s direct regulation of longevity-associated factors, RHEB emerges as a terminal and functionally pivotal node mediating the anti-aging effect of ACP in spleen tissue. To evaluate the translational potential of this finding, the conservation of RHEB between mice and humans was examined. DNA sequences of RHEB were highly conserved, exhibiting 98.91% amino acid identity and relatively conserved amino acid sequence of RHEB protein. Structural dynamics analysis revealed that RMSF values across the four chains of the human-derived RHEB remained below 2.0 Å, indicating overall conformational stability, though limited regions exhibited higher fluctuations, likely reflecting local flexibility (Ameji et al., 2023). Residue interaction maps, dominated by chartreuse-colored regions (scale <1.0), suggested a prevalence of moderate inter-residue contacts (Ameji et al., 2023). Further assessments of molecular stability (Yazdani et al., 2024), including deformability, B-factor, eigenvalue, variance and covariance matrices, and elastic network modeling collectively indicated that RHEB maintained structural integrity conducive to stable interactions with ACP. DockSurf analysis, which maps interaction landscapes based on biomolecular orientation (Barbault et al., 2023), generated thermodynamic profiles (ΔH_MM_, ΔH_QM_, ΔSolv, ΔG_MM_, ΔG_QM_) supporting the physical plausibility of ACP-RHEB binding. These data underscore the high evolutionary and structural conservation of RHEB, supporting the translational relevance of RHEB-targeted strategies identified in murine models to potential clinical applications in humans.

Three independent molecular docking tools were employed to evaluate the binding potential between the four core ACP metabolites (glucuronic acid, anhydrous glucose, galactose, arabinose) and the human RHEB. Analysis revealed that the resulting complexes were stabilized predominantly by conventional hydrogen bonds, van der Waals interactions, and carbon-hydrogen bonds. The calculated binding energies for ACP metabolite-RHEB pairs were consistently below −4 kcal/mol, indicating robust binding affinity (Liu et al., 2026). These findings were aligned with the molecular glue hypothesis, in which small molecules facilitate target protein ubiquitination and degradation by bridging them to E3 ubiquitin ligases (Hemant Kumar et al., 2024). Consistent with this model, ACP treatment significantly increased the protein levels of three E3 ligases including RNF185 and RNF121 in aged spleen tissue. Subsequent docking simulations confirmed stable, high-affinity binding between ACP metabolites and these human-derived ligases, with energies below −5 kcal/mol. These data support a mechanistic model wherein ACP metabolites may simultaneously engage RHEB and specific E3 ligases, promoting RHEB ubiquitination and degradation. This would lead to downstream inhibition of the mTOR pathway, contributing to the attenuation of spleen aging. This mechanism may also explain why RHEB downregulation was significant at protein level, but not its transcripts, in the present multi-omics sequencing work.

### 4.5 ACP-mediated senescence alleviation via RHEB inhibition

Integrated with the present experimental data, the analysis demonstrated that ACP significantly mitigated cellular senescence, an effect mediated, at least in part, through inhibition of the RHEB/mTORC1 signaling axis and closely mirroring the outcomes of RHEB knockdown. Specifically, ACP ameliorated three core hallmarks of cellular decline, including the accumulation of ROS, increased apoptosis, and the induction of a senescent phenotype. Mechanistically, the ability of ACP to reduce intracellular ROS likely originates from its suppression of RHEB-mediated mTORC1 hyperactivation, as aberrant mTORC1 signaling is known to enhance mitochondrial oxidative metabolism and drive excessive ROS production (Tang et al., 2019). The concomitant reduction in splenic apoptosis may also be linked to this decrease in ROS, given the central role of ROS in regulating mitochondrial, death receptor, and endoplasmic reticulum stress-mediated apoptotic pathways (Redza-Dutordoir and Averill-Bates, 2016). Furthermore, the decrease in SA-β-gal-positive cells following ACP treatment or RHEB interference provided direct evidence of the potent anti-senescence activity of ACP. Since both ROS and mTORC1 signaling are intimately connected to the establishment and maintenance of cellular senescence, ACP may function by interrupting this pro-senescence cycle, thereby helping to preserve the proliferative capacity and functional integrity of cell populations. This conceptual framework, which applies to both aging and cancer, has recently been integrated into modern anti-aging and senotherapeutic strategies, including the use of senomorphics (agents that suppress the detrimental SASP) and senolytics (agents that selectively eliminate senescent cells) (Kusmanto, 2025). This can be attributed to chemotherapy and radiation, which can induce therapy-induced senescence in cancer cells, initially acting as a barrier against tumor progression. However, the accumulation of these therapy-induced senescent cells is a significant obstacle to long-term efficacy due to the sustained secretion of SASP factors, which remodel the tumor microenvironment to promote inflammation, angiogenesis, and therapy resistance and cancer recurrence (Kusmanto, 2025). Integrated analysis demonstrates that ACP reduces SA-β-gal positivity, ROS levels, and the expression of the pro-inflammatory mediators TNFAIP2 and CXCR3. Considering the above-mentioned functions of TNFAIP2 and CXCR3, ACP is proposed to disrupt the positive feedback loop between inflammation and senescence. Pharmacologically, the absence of increased cell death and the predominant suppression of senescence-associated inflammatory markers suggest that ACP acts primarily as a senomorphic agent, ameliorating the senescent phenotype and the associated secretory profile. This senomorphic property, coupled with its ability to downregulate RHEB/mTORC1 signaling, positions ACP as a promising natural product for mitigating aging.

### 4.6 Reversal of aging-associated gene signature concurrently attenuating senescence and tumorigenesis

Consistent with aging-associated dysregulation, the expression of *ABI2*, *ASPM*, and *SKA1* was significantly upregulated, while *FOXO1* was downregulated, in lymphoma samples from the GSE57520 and GSE25550 datasets. These protein expression patterns were mirrored in the MG compared to CG, and were reversed following ACP intervention in the AG. Unlike its unclear roles in aging, upregulation of ABI2 occurs in diverse cancers, including hepatocellular carcinoma and acute myeloid leukemia, and is correlated with adverse prognostic outcomes (Chen et al., 2022; Tao et al., 2025). ASPM, whose gene expression is upregulated across most cancer types and demonstrates a significant positive correlation with tumor aggressiveness, drives malignant progression and ultimately reduces the life expectancy of cancer patients (Liu et al., 2022b). Cellular aging correlates with dysregulation of spindle assembly and abnormal spindle formation driven by ASPM transcriptional upregulation (Zhang et al., 2023). A pan-cancer analysis demonstrated that SKA1 is overexpressed across diverse cancer types, and its elevated expression is significantly associated with reduced overall survival and increased recurrence (Lan et al., 2023). Pathophysiological factors in type 2 diabetes, including high glucose, enhance SKA1 expression, leading to centrosome amplification and diabetes-specific aging (Liu et al., 2019; Zhang et al., 2025). Against the backdrop of tumor-associated oxidative stress and PI3K/AKT hyperactivation, FOXO1 is more frequently observed as a tumor suppressor that is inhibited, and such inhibition is associated with tumor progression (Guan et al., 2025). FOXOs, including FOXO1, are implicated as contributors to extreme longevity and healthspan (Martins et al., 2016). Therefore, by downregulating ASPM, SKA1 while upregulating FOXO1, ACP may concurrently attenuate aging processes and inhibit tumor.

### 4.7 Limitations and future directions

The present study primarily focused on the spleen as the organ of interest, given its unique role as the largest secondary lymphoid organ and blood filter, as well as its high vascularity and direct exposure to circulating inflammatory and senescent stimuli. Whereas, anatomically, the splenic artery originates from the celiac trunk and courses within the splenorenal ligament, laterally and across the superior aspect of the pancreas (Omole and Launico, 2026). Natural plant polysaccharides such as ACP, when administered orally, are absorbed into the bloodstream and may exert systemic effects beyond the spleen. The current study did not systematically evaluate these potential off-target effects, which represents a limitation of the current work. Therefore, future investigations should adopt a multi-organ approach to comprehensively assess the biodistribution, pharmacokinetics, and tissue-specific effects of ACP. Also, the present work was to validate the protective effects of ACP on the aging spleen using a well-established reference dose. However, the absence of testing a gradient of doses is a limitation. A dose-response design would have allowed us to determine the minimum effective dose, identify dose-dependent responses, and identify potential toxicity thresholds. Future studies should incorporate multiple dose levels to establish an optimal therapeutic regimen and to fully characterize the pharmacological profile of ACP. While the SAMP8 model justifies the use of six-month-old mice, the accelerated aging phenotype may not fully replicate all aspects of natural aging observed in older normally aging strains.

## 5 Conclusion

The present study delineated a coherent molecular framework through which ACP ameliorate spleen aging. Using a multi-omics approach, RHEB was identified as a key node in ACP-mediated anti-aging effects. In aged mice, ACP reduced splenic apoptosis and inflammation, and downregulated the expression of RHEB, a key activator of mTORC1. Structural and docking analyses demonstrated that ACP metabolites bond stably to human RHEB and to several E3 ubiquitin ligases that were upregulated upon ACP treatment, supporting a molecular glue-like mechanism that may promote RHEB ubiquitination and degradation. Moreover, ACP reversed the age-associated expression of several genes linked to both senescence and tumorigenesis via downregulating ASPM, SKA1 while upregulating FOXO1, highlighting a potential dual role in decelerating aging and inhibiting cancer-related pathways. Collectively, these findings positioned ACP as a multifaceted agent that attenuated splenic aging through RHEB/mTORC1 inhibition, likely via ubiquitin-mediated degradation, while simultaneously modulating a expression network of aging-associated genes. This work not only deepened the mechanistic understanding of how botanical polysaccharides counteract aging but also established a translational framework for targeting RHEB-mediated pathways in age-related disorders.

## Data Availability

The raw sequencing data have been deposited in the China National GeneBank Sequence Archive (CNSA) under accession number CNP0009902 (https://db.cngb.org/data_resources/project/CNP0009902).
